# Duloxetine reduces opioid consumption and pain after total hip or knee arthroplasty: a meta-analysis of randomized controlled trials

**DOI:** 10.1186/s13018-024-04648-5

**Published:** 2024-03-13

**Authors:** Yicai Lin, Mingyang Jiang, Chun Liao, Qingjian Wu, Jinmin Zhao

**Affiliations:** 1 Department of Bone and Joint Surgery, The First Affiliated Hospital of Guangxi Medical University, Guangxi, China; 2 Collaborative Innovation Centre of Regenerative Medicine and Medical BioResource Development and Application Co-constructed by the Province and Ministry, Guangxi Medical University, Nanning, Guangxi 530021 China; 3 Department of Trauma Orthopedic and Hand Surgery, The First Affiliated Hospital of Guangxi Medical University, Guangxi, China

**Keywords:** Total knee arthroplasty, Total hip arthroplasty, Duloxetine, Meta-analysis

## Abstract

**Purpose:**

There is no consensus in the current literature on the analgesic role of duloxetine after total hip arthroplasty (THA) or total knee arthroplasty (TKA). Thus, we designed this meta-analysis to reveal the analgesic effectiveness and safety of duloxetine in TKA or THA.

**Methods:**

As of October 2022, two authors (L.C. and W.Q.J.) independently searched five main databases (EMBASE, Web of Science, PubMed, Cochrane Library, and Google Scholar) to find relevant studies. Duloxetine vs. placebo in randomized controlled trials (RCTs) for THA or TKA were included. We set perioperative total opioid consumption as the primary outcome. Secondary outcomes included resting or dynamic pain scores over time, gastrointestinal adverse events, neurological adverse events, and other adverse reactions.

**Results:**

Eight RCTs with 695 patients were incorporated in our study. This meta-analysis showed high evidence that duloxetine was effective in reducing perioperative opioid consumption (Standard mean difference [SMD] = − 0.50, 95% confidence intervals [CI]: −0.70 to − 0.31, *P* < 0.00001) and low to moderate evidence that duloxetine could reduce pain within three weeks after surgery. Low to high evidence showed no differences between the two groups for most adverse events. Substantial evidence suggests that duloxetine can reduce nausea and vomiting after surgery (Risk ratio [RR] = 0.69, 95% CI: 0.50 to 0.95, *P* = 0.02, I^2^ = 4%). However, moderate evidence suggested that duloxetine might be associated with increased postoperative drowsiness (RR = 1.83, 95% CI: 1.08 to 3.09, *P* = 0.02, I^2^ = 0%).

**Conclusion:**

Duloxetine reduced overall opioid consumption in the perioperative period and relieved pain within three weeks after surgery without increasing the risk of adverse drug events. Duloxetine can be part of a multimodal management regimen in patients with THA and TKA.

**Supplementary Information:**

The online version contains supplementary material available at 10.1186/s13018-024-04648-5.

## Introduction

The primary treatment options for patients with end-stage degenerative arthritis are total hip arthroplasty (THA) and total knee arthroplasty (TKA), which can effectively reduce chronic pain and enhance joint function. Given the incredible trauma of these two types of procedures, many patients are dissatisfied with postoperative pain [1, 2]. Inadequate pain control delays recovery, prolongs hospital stays, and increases the risk of postoperative complications. At present, opioid drugs are still widely used in perioperative and postoperative pain control [3]. Opioids relieve pain but can cause nausea, vomiting, constipation, drowsiness, and other adverse effects [4]. Furthermore, overdependence on opioid drugs for pain management is related to opioid dependence and hyperalgesia [5]. These all bring great troubles to patients’ postoperative lives. Multimodal analgesia is designed to use combinations of multiple drugs or techniques to lower the dose of each drug, thereby reducing the side effects of each drug while maintaining overall efficacy [6, 7]. In theory, it could reduce opioid consumption, reduce pain, and reduce opioid-related adverse reactions [8].

Duloxetine was initially used to treat major depressive disorder and was later expanded to treat fibromyalgia, chronic musculoskeletal pain, and diabetic peripheral neuropathy [9–12]. The downward inhibitory pain pathway in the central nervous system can be suppressed by duloxetine, which inhibits the uptake of serotonin and norepinephrine [13–15]. Recent evidence suggests that duloxetine may help mitigate the acute central sensitization associated with post-surgical tissue injury [16]. In individuals with centrally mediated musculoskeletal pain, duloxetine is effective as an analgesic [17, 18]. Therefore, duloxetine can theoretically relieve pain after various surgical procedures.

There is no consensus in the current literature on the analgesic role of duloxetine after TKA or THA. Some studies suggest that although duloxetine has an opioid-sparing effect in the perioperative period of TKA, it does not bring an additional analgesic effect [19, 20]. However, studies also support its analgesic advantage over a placebo in TKA or THA [21, 22]. Therefore, this systematic review and meta-analysis were designed to validate the analgesic curative effect and security of duloxetine in TKA and THA.

## Materials and methods

This study was structured to adhere to the AMSTAR and PRISMA, which consist of mandatory specifications for open data reporting [23]. We followed a standard technique already registered on the PROSPERO (CRD42023403471). Since this meta-analysis relied only on already-published papers, no ethical clearance was required.

### Search strategy

As of October 2022, two authors (L.C. and W.Q.J.) independently searched EMBASE, Google Scholar, PubMed, Cochrane Library, and Web of Science using the following keywords: (TKR or total knee replacement TKA or total knee arthroplasty or THR or total hip replacement or THA or total hip arthroplasty) and (duloxetine or cymbalta) to find the relevant material. Further omissions were prevented by hand-checking references and citations of potentially relevant material.

### Eligibility criteria for study selection

According to the PICOS concept, the following inclusion criteria were established for the pertinent research in this paper: (1) patients who received THA or TKA; (2) the intervention group received duloxetine before and after surgery; (3) the control group received placebo before and after surgery; (4) there are indicators related to analgesia in the results, such as opioid consumption, pain score, and adverse event; (5) randomized controlled trials (RCTs) in English journals. Exclusion criteria included: (1) exclusion of non-English articles, reviews, editorials, letters, case reports, duplicate publications, conference abstracts, and guidelines; (2) studies using duloxetine and placebo only before or after surgery; (3) studies with no analgesic-related indicators or no extractable data in the results; (4) patients with trauma, cognitive impairment, alcoholism, and drug abuse. All references included in this study were rigorously screened by the inclusion and exclusion criteria established above. Negotiation or consultation with third authors was used to resolve differences between two independent authors in literature screening. The Kappa value was utilized to determine the degree to which the two authors agreed throughout the article screening process.

### Quality assessment

All included studies were independently evaluated for bias by two of our authors (L.C. and W.Q.J.) using Cochrane’s risk-of-bias tool for randomized trials (RoB2) and the criteria specified in the Cochrane Handbook for Systematic Reviews of Interventions [24]. The writers (L.C., J.M.Y., and W.Q.J.) discussed and reached a consensus on how to handle disagreements. The risk of bias for each item was categorized as low, high, or uncertain (deficiency of data or unidentified risk of bias) based on the information supplied by the included studies. The following are the calculated Kappa values that assess the level of consensus amongst authors (L.C. and W.Q.J.): fair (0.40 to 0.59), good (0.60 to 0.74), and excellent (0.75 or more).

### Data collection

Applying standardized data extraction documents, two writers (L.C. and J.M.Y.) independently completed the data extraction task. The subsequent information was taken from the included studies: first author, publication year, sample size, sex ratio, average age, intervention, operation type, dosage, and timing of duloxetine, outcome measures, follow-up time, etc. The corresponding authors of the papers that lacked necessary data for meta-analysis or had just visual data presentation were contacted via email. If that wasn’t the case, we followed the protocols outlined in the Cochrane Handbook for Systematic Reviews of Interventions. If it became essential, we would stop extracting partial data. Disagreements arising from data collecting were settled via open dialogue.

### Primary and secondary outcomes

Considering the subjectivity of pain scores, we set perioperative total opioid consumption as the primary outcome. Secondary outcomes include resting or dynamic pain scores over time, gastrointestinal adverse events, neurological adverse events, and other adverse effects.

### Quality of evidence

The GRADE method was utilized to evaluate the strength of evidence for pooled results [25]. We downgraded the outcomes based on the risk of bias, indirectness, inconsistency, impreciseness, and publication bias of the contained literature.

### Statistical analysis

Data were analyzed by RevMan (version 5.4.0) and Stata (version 14.0). Risk ratio (RR) with a 95% confidence interval (CI) or standardized mean difference (SMD) with 95% CI were utilized to assess dichotomous or continuous consequences, respectively. Heterogeneity was evaluated using Begg’s and Egger’s tests and the I^2^ statistic. I^2^ < 25% was chosen to indicate low heterogeneity and I^2^ > 75% was selected to indicate high heterogeneity. The fixed effect model was used when the I^2^ value was < 50%, while the random effect model was used when I^2^ was > 50%. Significance was set at *P* < 0.05. Sensitivity analysis was performed to examine the stability of meta-analysis results. Considering the possible causes for heterogeneity, subgroup analyses were performed for the prospective outcomes depending on the anesthesia strategy, type of surgery, dosage of duloxetine, and risk of bias.

## Results

### Search results and study characteristics

**Fig. 1:**
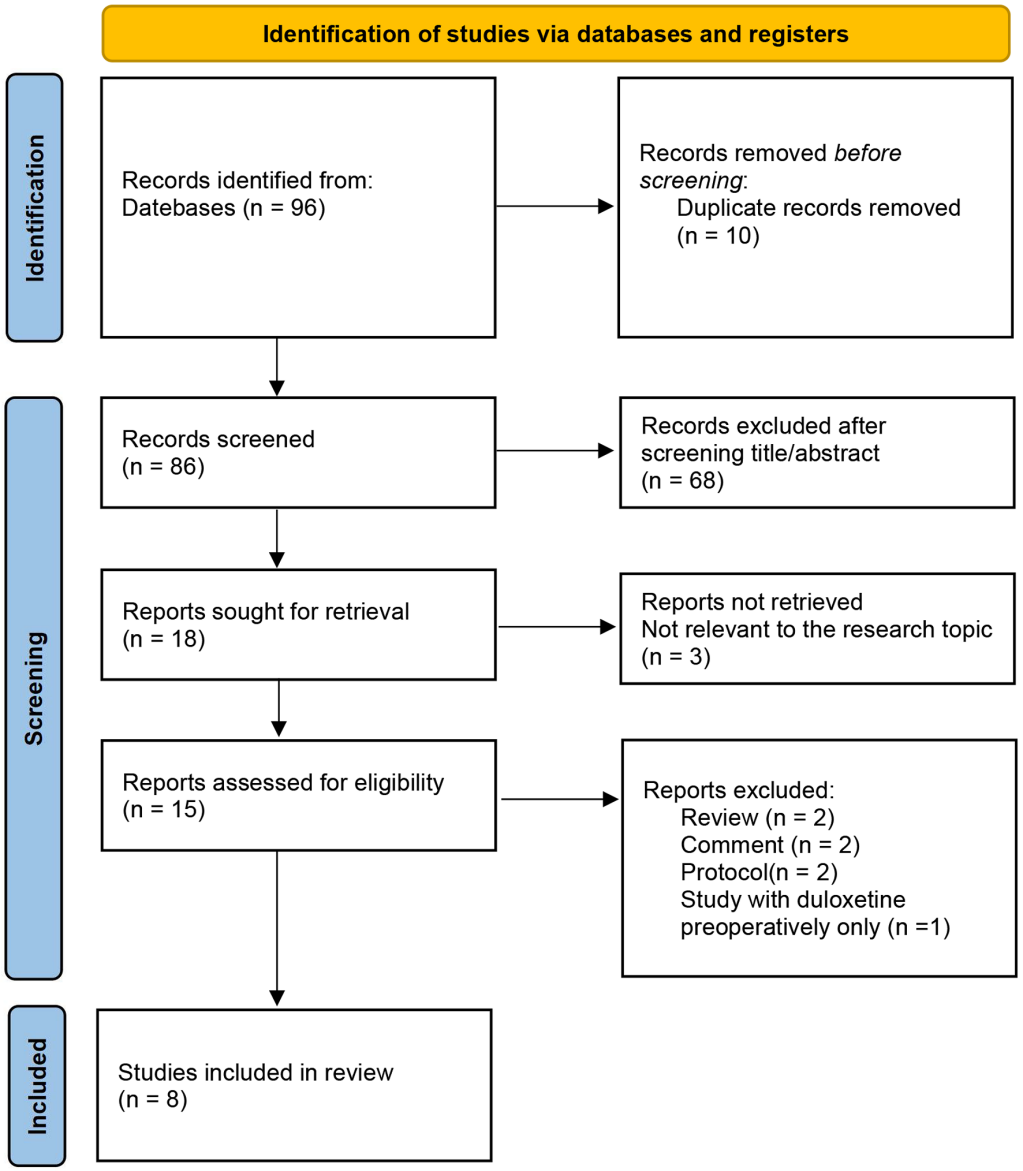
PRISMA Flow diagram

The PRISMA Flowchart illustrates the steps used to appraise appropriate studies included in the literature (Fig. 1). After eliminating duplicate studies, 86 articles were included in the screening. According to screening the title and abstract, 18 articles were included in this study. Finally, 8 full-text publications passed the first screening and were ultimately analyzed [19–22, 26–29].

The 8 RCTs investigated a total of 695 people (Table 1). The sample size of the included studies ranged from 39 to 160, with a distribution of 347 in the duloxetine group and 348 in the control group. Regarding the surgical type, 6 involved TKA [19, 20, 22, 27–29], and 2 involved THA [21, 26].

**Table 1:** Characteristics of studies included in the meta-analysis

Author	Year	Country	Sample size		Women, No. (%)		Average age (years)		BMI		Intervention		Operative type	Follow-up	Endpoints
			E	C	E	C	E	C	E	C	E	C			
K.-Y. Ho1 [19]	2010	Singapore	23	24	16(70)	17(71)	65.2	65.7	N/A	N/A	60 mg of oral duloxetine 2 h before surgery and the first day after surgery	Duloxetine not used	TKA	6 months	opioid consumption, dynamic pain scores, AEs
Jacques T. Ya Deau [20]	2016	American	53	53	28(52.8)	26(49.1)	67	63	N/A	N/A	60 mg orally daily for 15 days, starting on the day of surgery	Duloxetine not used	TKA	3 months	Neasua and vomiting
In Jun Koh [28]	2019	South Korea	40	40	35 (88)	34(85)	69.1 ± 5.8	68.6 ± 9.5	25.5 ± 2.3	26.4 ± 7.5	30 mg of oral duloxetine on the night before surgery and 30 mg per day for 6 weeks after surgery	Duloxetine not used	TKA	12 Months	Resting pain scores, dynamic pain scores, AEs
Man Soo Kim [27]	2021	South Korea	19	20	17(89.5)	16(80)	71.2 ± 6.5	67 ± 7.1	25.7 ± 4.5	25.7 ± 3.4	30 mg per day from 2 weeks before surgery to 8 weeks after surgery	Duloxetine not used	TKA	3 months	Resting pain scores, dynamic pain scores, AEs
Hao Li [21]	2021	China	48	48	26(54)	24(50)	52.7 ± 12.0	50.2 + 13.2	24.0 ± 2.9	23.9 ± 3.4	60 mg of oral duloxetine every night since preoperative day 2 till postoperative day 14	Duloxetine not used	THA	3 months	opioid consumption, resting pain scores, dynamic pain scores, AEs
Zi-chuan Ding [26]	2022	China	34	33	21(61.8)	18(54.5)	58	61	23.8	23.6	60 mg of oral duloxetine from the day of surgery to postoperative day 6	Duloxetine not used	THA	3 months	AEs
Jacques T. Ya Deau [22]	2022	America	80	80	40(50)	35(44)	63 ± 11	64 ± 7	31 ± 8	30 ± 7	60 mg of oral duloxetine on the day of operation and 60 mg per day for 2 weeks after surgery	Duloxetine not used	TKA	3 Months	opioid consumption, resting pain scores, dynamic pain scores, AEs
Mingcheng Yuan [29]	2022	China	50	50	30 (60)	27 (54)	67.8 ± 10.12	66.2 ± 9.83	24.67 ± 4.35	24.83 ± 3.87	60 mg of oral duloxetine every night since preoperative day 2 till postoperative day 14	Duloxetine not used	TKA	3 Months	opioid consumption, resting pain scores, dynamic pain scores, AEs

*Note:* E, experimental group; C, control group; AEs, adverse events

### Risk of bias

**Fig. 2:**
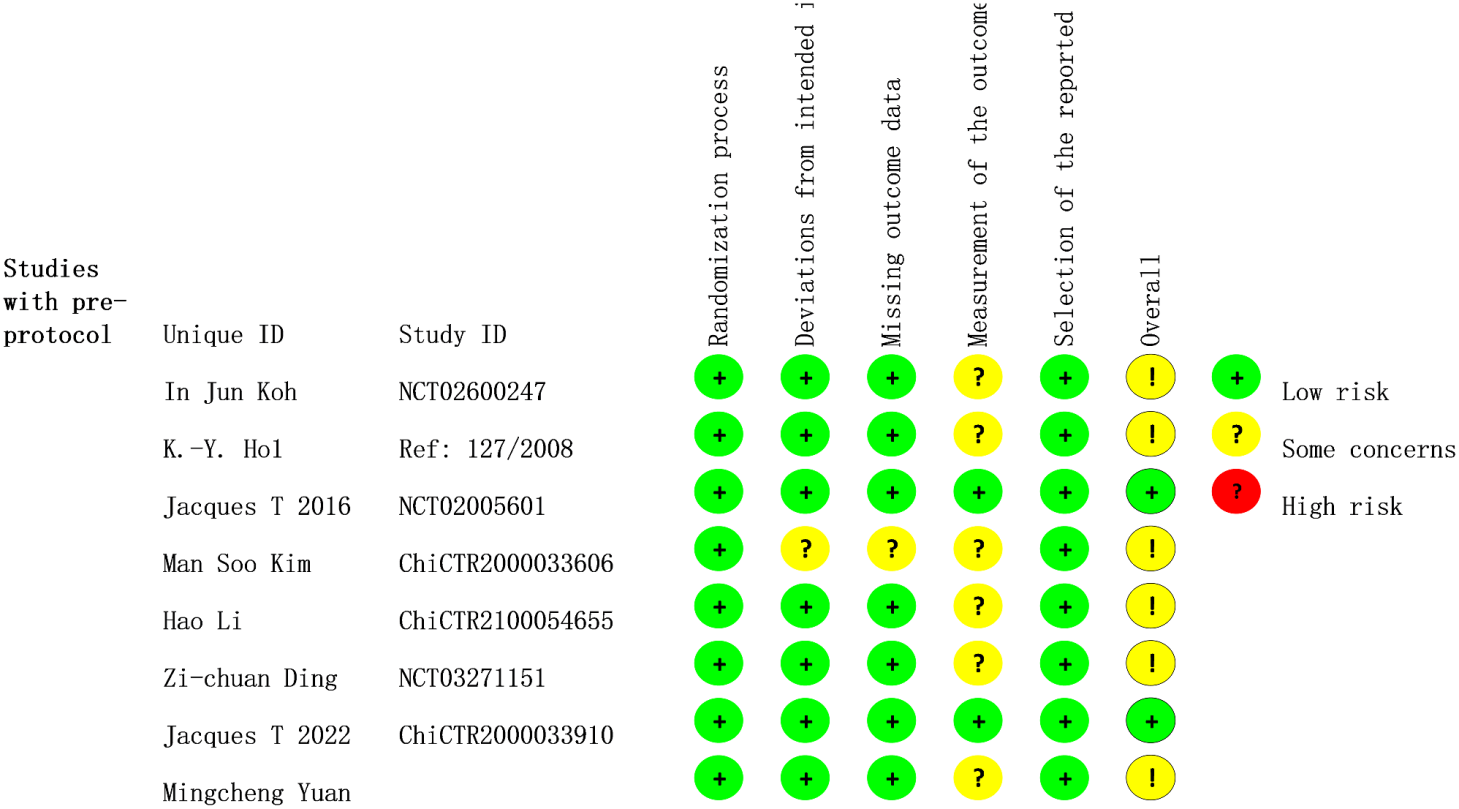
Results of Cochrane’s risk-of-bias tool for randomized trials (RoB2)

All included research represented the approach of randomization and allocation concealment. The vast majority of studies emphasized blinding of patients, implementers, and data collectors. A standardized assessment of the risk of bias in the 8 studies was summarized in Table 2. The evaluation findings of the RoB2 tool are displayed in Fig. 2, where two studies indicate low risk and the remaining studies indicate some concerns [20, 22]. Overall, there was an outstanding level of agreement between the two reviewers (kappa = 0.714) when it came to assessing the risk of bias (Table A.2).

**Table 2:** Assessment of methodological quality of included studies

Study	Random allocation	Hidden distribution	Blind method	Incomplete Outcome Data	Selective reporting of results	Other bias	Quality grade
K.-Y. Ho 2010	Randomized	No clear	Double-blind	Low	Low	Low	B
Jacques T. YaDeau 2016	Randomized	No clear	Triple-blind	Low	Low	Low	A
In Jun Koh 2019	Randomized	No clear	Triple-blind	Low	Low	Low	A
Man Soo Kim 2021	Randomized	No clear	Double-blind	Low	Low	Low	B
Hao Li 2021	Randomized	No clear	Double-blind	Low	Low	Low	B
Zi-chuan Ding 2022	Randomized	No clear	Double-blind	Low	Low	Low	B
Jacques T. YaDeau 2022	Randomized	No clear	Triple-blind	Low	Low	Low	A
Ming cheng Yuan 2022	Randomized	No clear	Double-blind	Low	Low	Low	B

### Primary outcome (total opioid consumption)

**Fig. 3:**
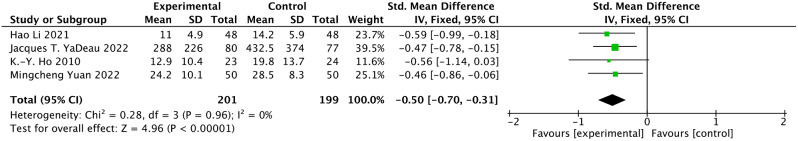
Forest plots of the total opioid consumption using the fixed model

Total opioid consumption is shown in four studies [19, 21, 22, 29]. A total of 400 patients (experimental group = 201 and control group = 199) were involved in evaluating total opioid consumption. The evidence quality was highly certain for total opioid consumption (Table 3). The total opioid consumption of the duloxetine group was significantly lower (SMD = − 0.50, 95% CI: −0.70 to − 0.31, *P* < 0.00001, I^2^ = 0%) (Fig. 3).

**Table 3:** The result of Begg’s and Egger’s test for outcomes

Outcomes	Begg’s test p-Value	Egger’s test p-Value
Opioids consumption	0.308	0.397
Pain scores during rest at 1w	1	0.687
Pain scores during rest at 2-3w	0.734	0.113
Pain scores during movement at 1w	0.308	0.253
Pain scores during movement at 2-3w	1	0.761
Appetite loss	1	/
Constipation	0.462	0.578
Nausea and vomiting	1	0.987
Insomnia	1	0.728
Drowsiness	0.308	0.228
Dizziness	1	0.684
Dry mouth	0.462	0.742
Fatigue	1	0.463

### Secondary outcomes

#### Pain score at rest

**Fig. 4:**
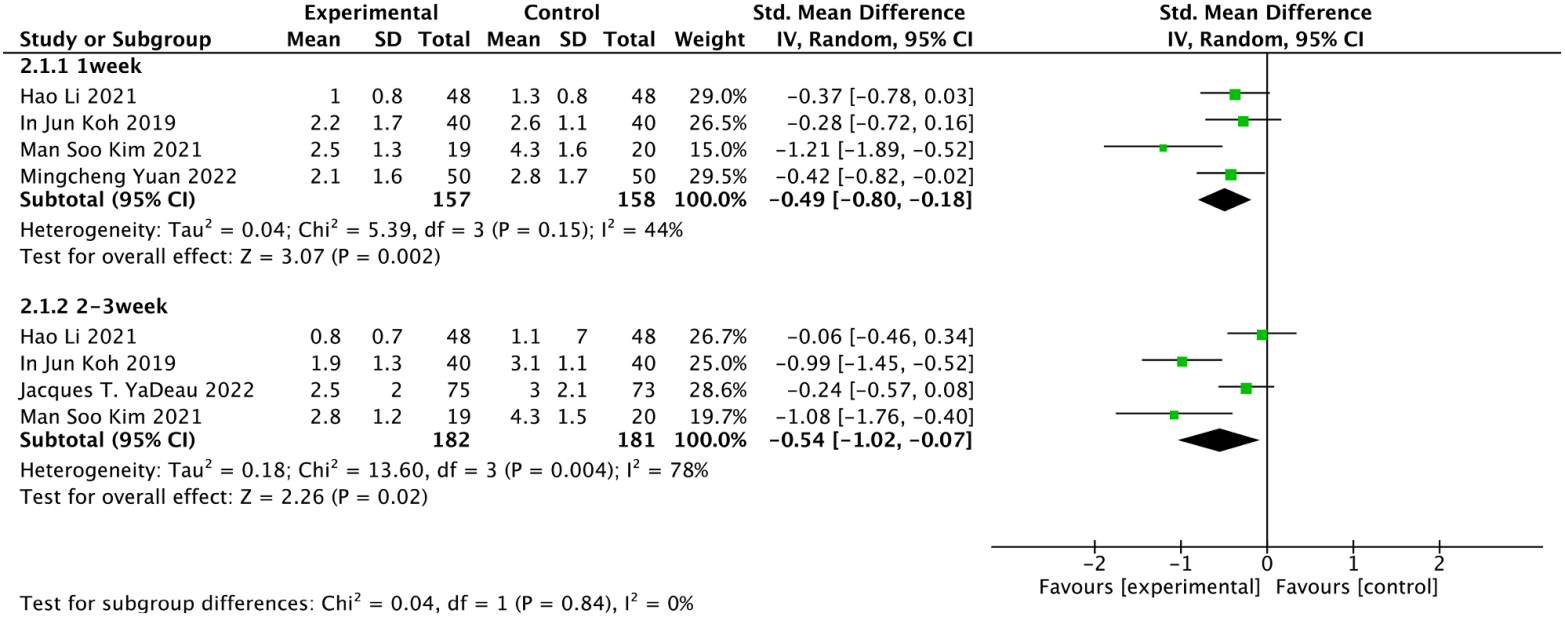
Forest plots of the pain score at rest using the fixed model

The pain score at rest is shown in the results of five articles [21, 22, 27–29]. The evidence quality was of moderate or low certainty for pain score at rest (Table 3). Meta-analysis of included studies suggested a significant pain improvement at rest in duloxetine groups vs. controls **(**SMD = − 0.49, 95% CI: −0.80 to − 0.18; *P* = 0.002, I^2^ = 44%) on the pain score at postoperative 1 week (including 315 patients), (SMD = − 0.54, 95% CI: −1.02 to − 0.07, *P* = 0.02, I^2^ = 78%) on the pain score at postoperative 2–3 week (including 363 patients) (Fig. 4).

### Pain score during movement

**Fig. 5:**
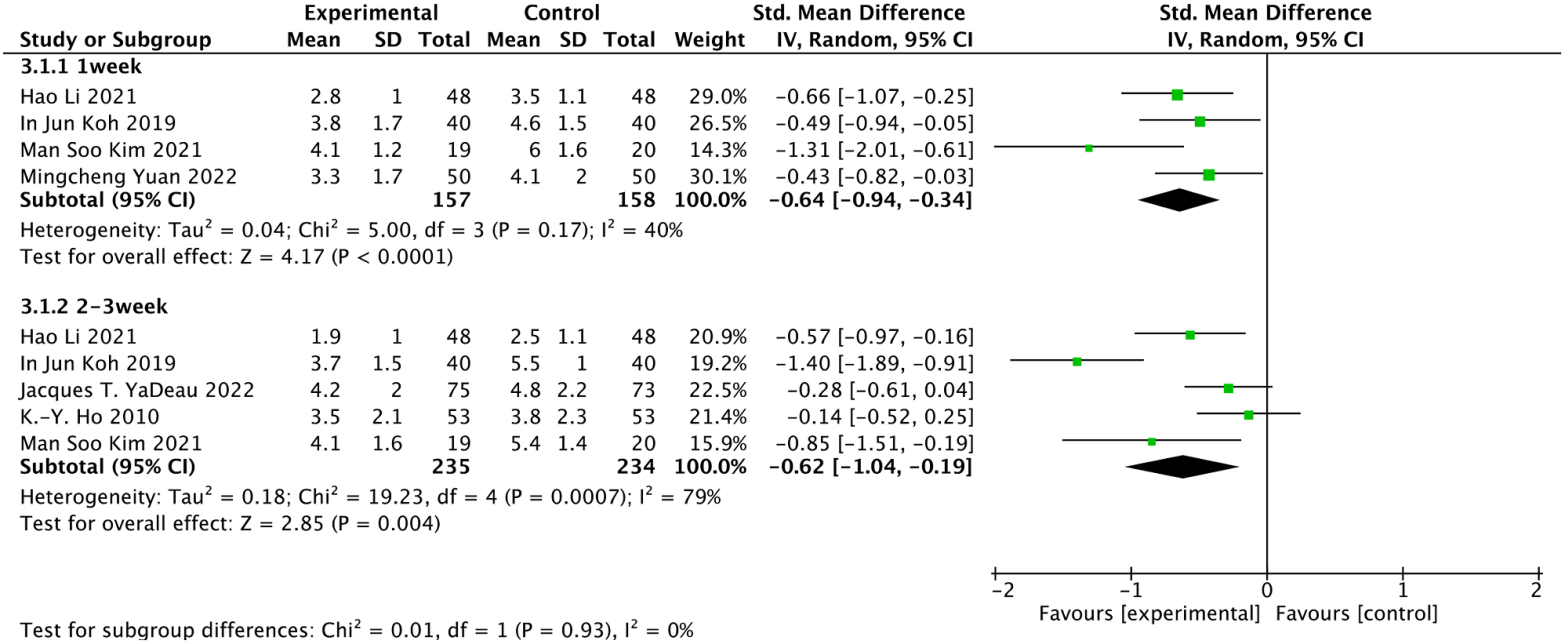
Forest plots of the pain score during movement using the fixed model

The pain score during movement is shown in the results of five articles [21, 22, 27–29]. We found that the quality of evidence was of moderate certainty for pain score during movement (Table 3). Meta-analysis of included studies suggested a significant pain improvement during movement in duloxetine groups vs. controls, (SMD = − 0.64, 95% CI: −0.94 to − 0.34, *P* < 0.0001, I^2^ = 40%) on the pain score at postoperative 1 week (including 315 patients), (SMD = − 0.62, 95% CI: −1.04 to − 0.19, *P* = 0.004, I^2^ = 79%) on the pain score at postoperative 2–3 week (including 363 patients) (Fig. 5).

### Gastrointestinal side effects (nausea and vomiting, constipation, and appetite loss)

**Fig. 6:**
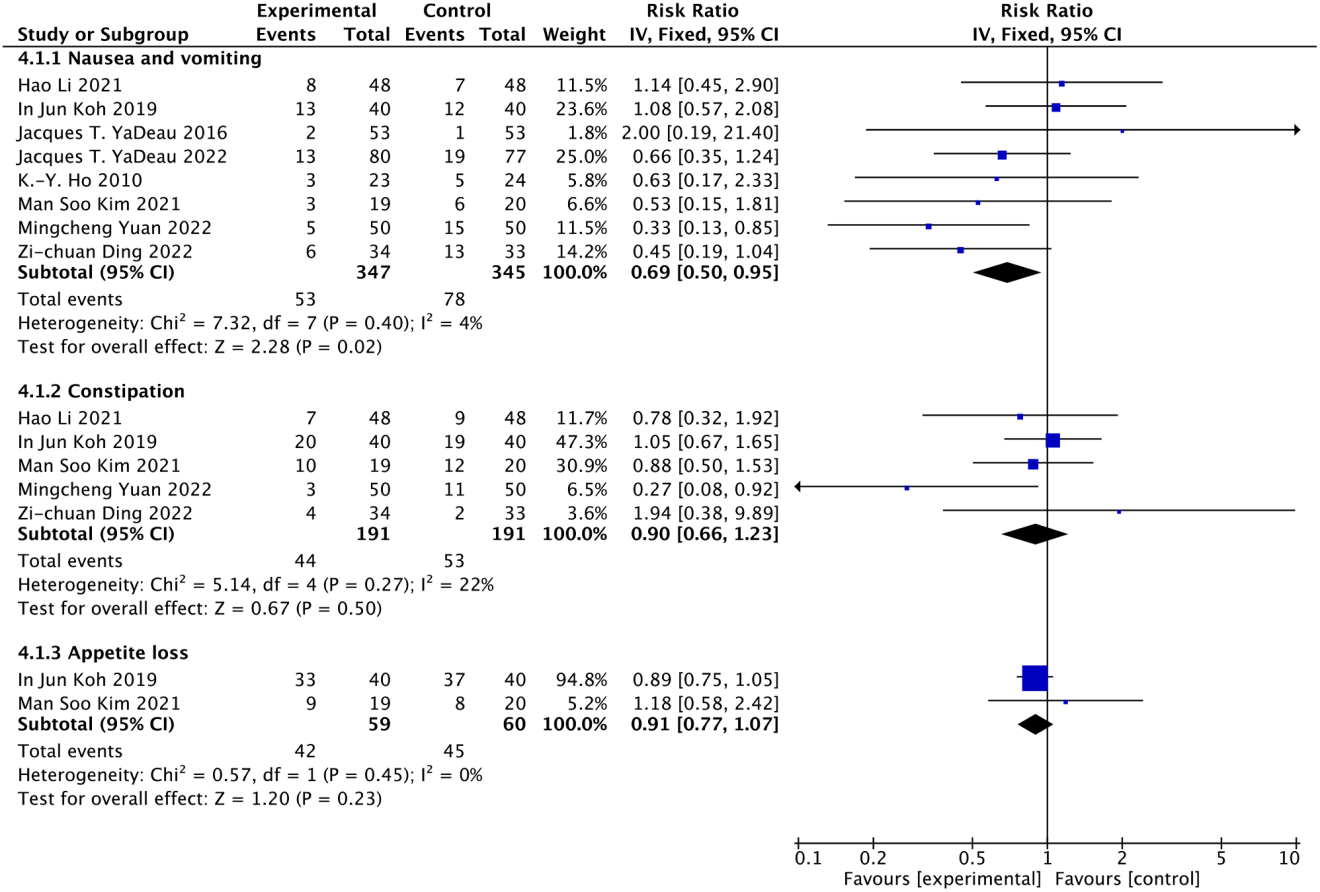
Forest plots of the gastrointestinal side effects using the fixed model

Eight articles reported nausea and vomiting [19–22, 26–29]. 692 people participated in the experiment, wherein 347 were classified to the duloxetine group, and 345 were classified to the control group. There is a significant difference in duloxetine groups vs. controls (RR = 0.69, 95% CI: 0.50 to 0.95, *P* = 0.02, I^2^ = 4%) on nausea and vomiting (Fig. 6). The quality of evidence is highly certain for nausea and vomiting (Table 3).

**Fig. 7:**
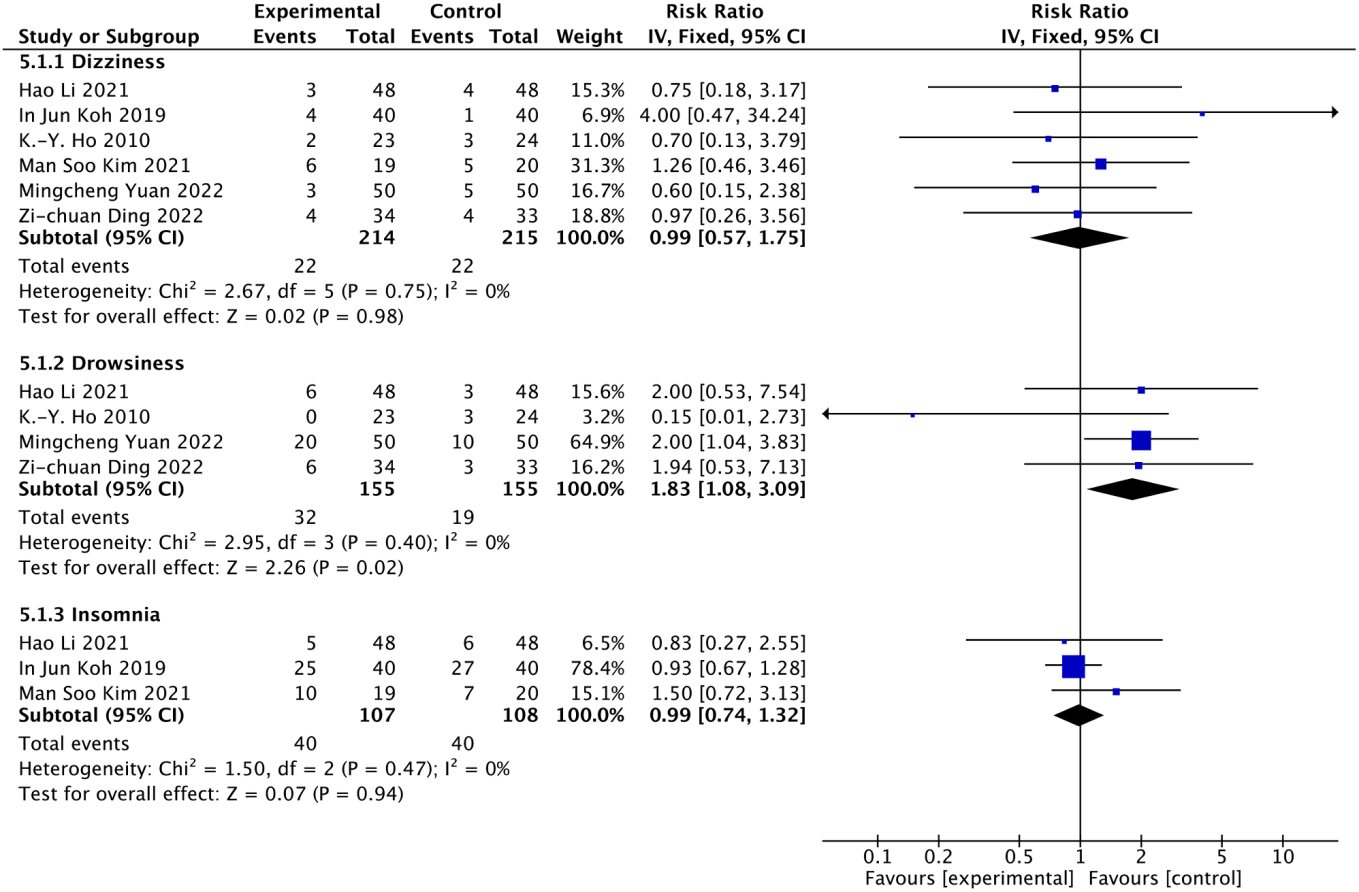
Forest plots of the nervous system side effects using the fixed model

Constipation is shown in the results of five articles [21, 26–29]. In the aggregate, 44 out of 191 patients in the duloxetine group suffered constipation. In the meantime, 53 out of 191 patients in the control group suffered constipation. Meta-analysis showed no difference in postoperative constipation between the duloxetine and control groups (RR = 0.90, 95% CI: 0.66 to 1.23, *P* = 0.50, I^2^ = 22%) (Fig. 7). The quality of evidence was of moderate certainty for constipation (Table 3). The consequence of the dosage subgroup revealed that duloxetine did not significantly reduce constipation compared to the control group when duloxetine was used in 30 and 60 mg doses (RR = 0.98, 95% CI: 0.69 to 1.39, *P* = 0.618, I^2^ = 0%; RR: 0.66, 95% CI: 0.34 to 1.29, *P* = 0.146, I^2^ = 48%) (Figure A.1.A).

Two articles reported appetite loss [27, 28]. In brief, 42 out of 59 patients in the duloxetine group underwent appetite loss. In the meanwhile, 45 out of 60 patients in the control group underwent appetite loss. Meta-analysis of included studies suggested a nonsignificant appetite loss in duloxetine groups vs. controls (RR = 0.91, 95% CI: 0.77 to 1.07, *P* = 0.23, I^2^ = 0%) (Fig. 6). The quality of evidence was of moderate certainty for appetite loss (Table 3).

### Nervous system side effects (dizziness, drowsiness, and insomnia)

Six articles reported dizziness [19, 21, 26–29]. 429 people participated in the evaluation, 214 were designated as the duloxetine group, and 215 were assigned as the control group. We found that the quality of evidence was highly certain for dizziness (Table 3). There was no significant difference in dizziness (RR = 0.99. 95% CI: 0.57 to 1.75; *P* = 0.98; I^2^ = 0%) (Fig. 7). The result of the dosage subgroup revealed that duloxetine did not significantly reduce dizziness when duloxetine was used in 30 and 60 mg doses (RR = 1.56, 95% CI: 0.62 to 3.87, *P* = 0.341, I^2^ = 0%; RR: 0.75, 95% CI: 0.37 to 1.54, *P* = 0.967, I^2^ = 0%) (Fig. 9, Figure A.1.B).

**Fig. 8:**
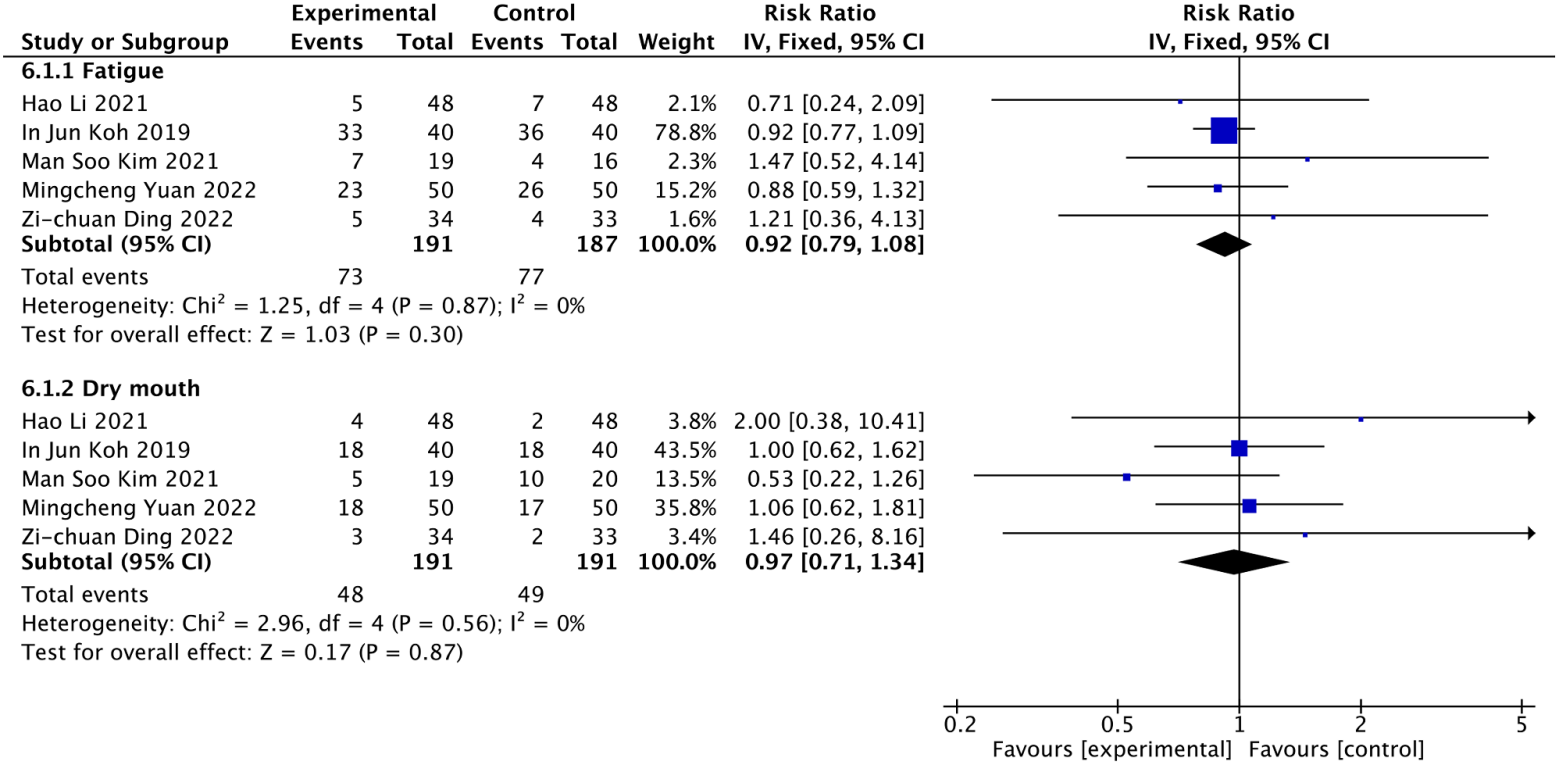
Forest plots of the fatigue and dry mouth using the fixed model

Drowsiness is shown in the results of five articles [19, 21, 26, 29]. 310 people participated in the evaluation, 155 were assigned to the duloxetine group, and 155 were assigned to the control group. The quality of evidence was of low certainty for drowsiness (Table 3). Meta-analysis of 5 studies showed a higher proportion of drowsiness in the duloxetine group vs. control (RR = 1.83, 95% CI: 1.08 to 3.09, *P* = 0.02, I^2^ = 0%) (Fig. 7).

Insomnia is shown in the results of three articles [21, 27, 28]. 215 people participated in the evaluation, 107 were assigned to the duloxetine group, and 108 were assigned to the control group. There was no significant difference in insomnia (RR = 0.99, 95% CI: 0.74 to 1.32, *P* = 0.07, I^2^ = 0%) (Fig. 7). The quality of evidence was of moderate certainty for insomnia (Table 3).

### Other adverse effects (dry mouth, fatigue)

Five articles reported dry mouth [21, 26–29]. 392 people participated in the evaluation, 191 were assigned as the duloxetine group, and 191 were designated as the control group. There is no significant difference in the dry mouth (RR = 0.97, 95% CI: 0.71 to 1.34; *P* = 0.87; I^2^ = 0%) (Fig. 8). The quality of evidence was of moderate certainty for dry mouth (Table 3).

**Fig. 9:**
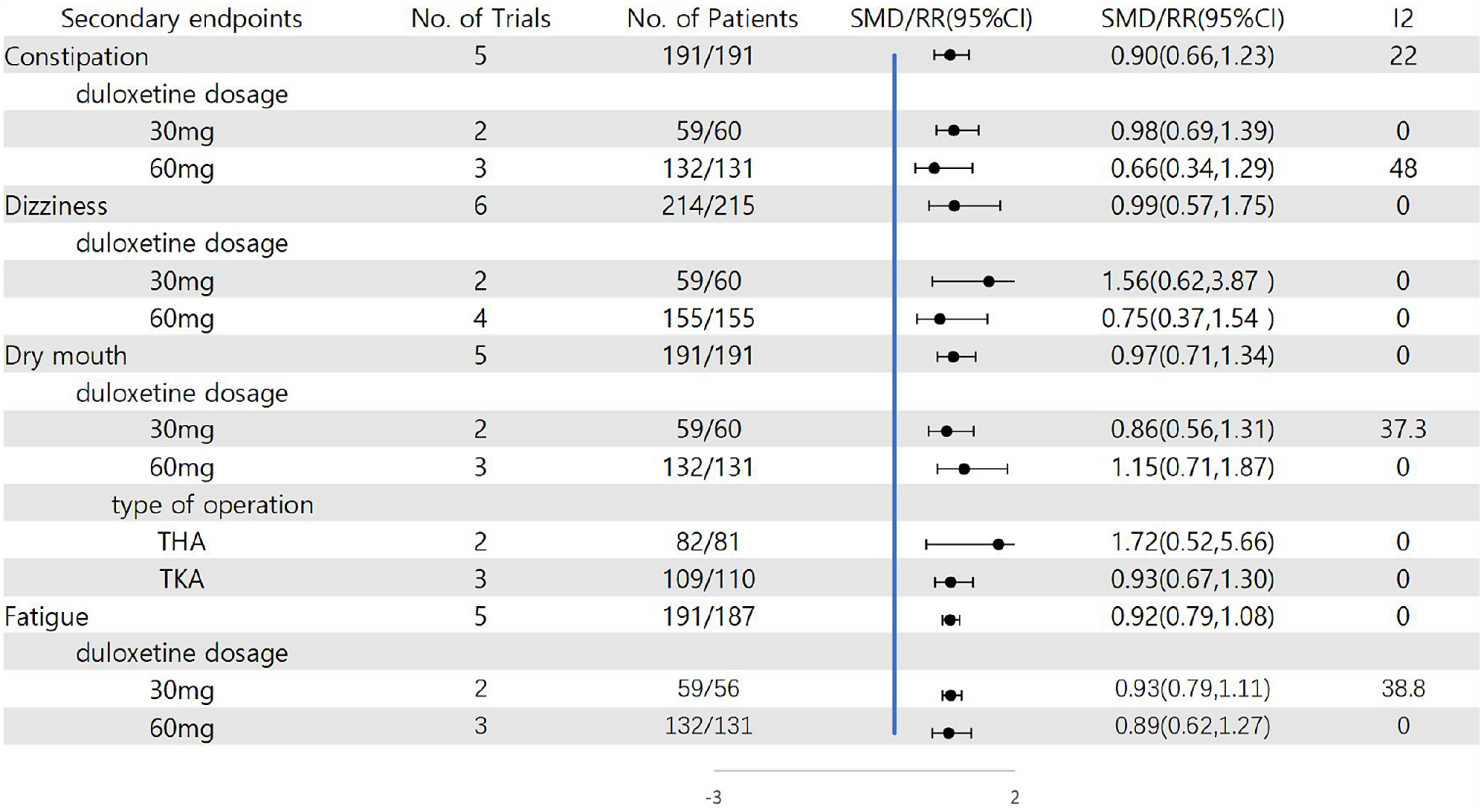
Result of subgroup analysis

Subgroup analysis was implemented based on the dosage of duloxetine. The consequence of the dosage subgroup revealed that duloxetine did not significantly reduce dry mouth when duloxetine was used in 30 and 60 mg doses (RR = 0.86, 95% CI: 0.56 to 1.31, *P* = 0.207, I^2^ = 37.3%; RR: 1.15, 95% CI: 0.71 to 1.87, *P* = 0.742, I^2^ = 0%) (Fig. 9, Figure A.1.C).

The consequence of the operation type subgroup revealed that duloxetine did not significantly reduce dry mouth when undergoing THA and TKA (RR = 1.72, 95% CI: 0.52 to 5.66, *P* = 0.794, I^2^ = 0%; RR: 0.93, 95% CI: 0.67 to 1.30, *P* = 0.376, I^2^ = 0%) (Fig. 9, Figure A.1.D).

Meta-analysis of 5 studies [21, 26–29] revealed no significant difference in fatigue with moderate certainty quality of evidence (RR = 0.92, 95% CI: 0.79 to 1.08; *P* = 0.30, I^2^ = 0%) (Fig. 8) (Table 3). The consequences of the dosage subgroup revealed that duloxetine did not significantly reduce fatigue when duloxetine was used in 30 and 60 mg doses (RR = 0.93, 95% CI: 0.79 to 1.11, *P* = 0.201, I^2^ = 38.8%; RR = 0.89, 95% CI: 0.62 to 1.27, *P* = 0.816, I^2^ = 0%) (Fig. 9, Figure A.1.E).

### Publication bias and sensitivity analysis

Publication bias in all outcomes was assessed according to Begg’s and Egger’s tests. The results show no significant publication bias for all outcomes (Table 4). Sensitivity analysis showed that the outcome of appetite loss was unstable. After removing the study by Koh et al. [28], the RR value of appetite loss fluctuated significantly. Other outcomes have good stability (Figure A.2.)

**Table 4:** GRADE evidence profile for outcomes

Outcomes	Relative effect (95% CI)	No of Participants (Studies)	Quality of the evidence (GRADE)
Total opioid consumption	-0.50 (-0.70, -0.31)	400 (4)	⊕⊕⊕⊕high
Pain score during rest at 1 week	-0.49 (-0.80, -0.18)	315 (4)	⊕⊕⊕moderate^2^
Pain score during rest at 2–3 week	-0.54 (-1.02, -0.07)	363 (4)	⊕⊕low^1, 2^
Pain score during activity at 1 week	-0.64 (-0.94, -0.34)	315 (4)	⊕⊕⊕moderate^2^
Pain score during activity at 2–3 week	-0.62 (-1.04, -0.19)	469 (5)	⊕⊕⊕moderate^1^
The rate of postoperative dizziness	0.99 (0.57, 1.75)	429 (6)	⊕⊕⊕⊕high
The rate of postoperative drowsiness	1.83 (1.08, 3.09)	310 (4)	⊕⊕low^1,2^
The rate of postoperative insomnia	0.99 (0.74, 1.32)	215 (3)	⊕⊕⊕moderate^2^
The rate of postoperative nausea and vomiting	0.69 (0.50, 0.95)	692 (8)	⊕⊕⊕⊕high
The rate of postoperative constipation	0.90 (0.66, 1.23)	382 (5)	⊕⊕⊕moderate^2^
The rate of postoperative appetite loss	0.91 (0.77, 1.07)	87 (2)	⊕⊕⊕moderate^2^
The rate of postoperative fatigue	0.92 (0.79, 1.08)	378 (5)	⊕⊕⊕moderate^2^
The rate of postoperative dry mouth	0.97 (0.71, 1.34)	382 (5)	⊕⊕⊕moderate^2^

^1^ Inconsistency (very high statistical heterogeneity exists, confidence interval overlap is small, and it cannot be explained by study design, differences in included populations, intervention methods, etc.); ^2^ Imprecision (the overall sample size is less than 400)

## Discussion

This meta-analysis revealed strong evidence that duloxetine effectively reduced perioperative opioid consumption and low to moderate evidence that duloxetine could improve pain levels within three weeks after surgery. There was low to high evidence that for most adverse events, such as constipation, dizziness, and fatigue, no differences were found between the two groups. Furthermore, high-level evidence showed that duloxetine could reduce postoperative nausea and vomiting. However, low-level evidence suggested that duloxetine might be associated with increased postoperative drowsiness.

There is a consensus in the pain community that tissue damage caused by surgery results in central and peripheral sensitization, and subsequent changes in neuroplasticity can lead to hyperalgesia in postoperative patients. The analgesic mechanism of duloxetine is through the modulation of serotonin and norepinephrine, thereby enhancing the descending inhibitory pain pathways in the brain and spinal cord and activating parts of the prefrontal lobe of the brain [13, 15, 30]. Some studies have also suggested that duloxetine has an antinociceptive effect by blocking Na^+^ channels and inhibiting neuronal cell firing caused by peripheral injury [31, 32]. Since TKA and THA are types of joint surgery with significant tissue trauma, many patients will experience mood changes after surgery, such as depression and insomnia. Therefore, it is essential to assess the effect of antidepressants such as duloxetine on the quality of recovery after TKA and THA.

Our results indicated that duloxetine had a significant advantage over the placebo. Pooling the primary outcome from four high-quality studies showed a significant opioid-sparing advantage for duloxetine, with no heterogeneity in the pooling outcome. In addition, duloxetine showed a sustained advantage within three weeks on pain scores at rest or during movement. Therefore, consistency in pain scores and opioid consumption reflects the stability of duloxetine analgesia after THA and TKA.

The optimal dose of duloxetine in lower extremity arthroplasty remains unclear. Of the studies we included, 6 studies used 60 mg daily [19–22, 26, 29], and 2 studies used 30 mg daily [27, 28]. Hetta et al. compared the analgesic effects of three preoperative doses (30, 60, and 90 mg) of duloxetine undergoing modified radical mastectomy [33]. We found that the overall quality of recovery was better for duloxetine 60 and 90 mg than for placebo and duloxetine 30 mg. However, no differences were observed on duloxetine 90 mg compared with those on 60 mg. By subgroup analysis of the secondary outcome, we found that 30 and 60 mg had no advantage over the placebo, and there was no statistical significance. In addition, we lack a dose grouping analysis of the main results. To sum up, the evidence found in this paper cannot infer the most effective dose of duloxetine. However, the use of 30 mg or 60 mg duloxetine has no adverse effect on the occurrence of postoperative complications. The best effective dose of duloxetine needs additional prospective studies to verify.

Regarding the safety of duloxetine, we selected several common adverse events as measurement indicators. The pooled outcomes found that the duloxetine group had a higher rate of drowsiness, while the incidence of nausea and vomiting was lower. However, no differences were found in other adverse events. It is a rather exciting finding since sleepiness, especially at night, may not be strictly a side effect. After all, proper sleepiness at night can effectively relieve anxiety and insomnia caused by surgery. From another perspective, improving sleep may improve postoperative pain. The view that serotonin is involved in vomiting has been inferred from its molecular biological function before the discovery of the serotonin selective regulation tool [34]. Thus, the inhibitory effect of duloxetine on 5-hydroxytryptamine reabsorption can well explain the outcome of duloxetine reducing postoperative nausea and vomiting. It is essential to recognize that duloxetine and opioids have a lot of overlapping effects. Thus, our pooled outcomes are superimposed on each other. In other words, reduced opioid consumption due to duloxetine can offset its adverse events. Therefore, we believe that duloxetine has a fairly solid safety profile.

Solving pain after total knee and hip arthroplasty remains a challenge. Recently, pregabalin has been considered an adjunctive medication for the treatment of neuropathic and postoperative pain. It has been used for postoperative analgesia in TKA and THA. Pregabalin is an antiepileptic drug that is structurally similar to GABA. It selectively affects the transmission pathway of pain in nociceptors by inhibiting calcium channels [35]. A meta-analysis report indicates that pregabalin has the effects of postoperative acute phase analgesia and reducing opioid consumption [36]. A double-blind clinical comparative trial has demonstrated that oral administration of pregabalin and duloxetine during the perioperative period can alleviate postoperative pain and reduce postoperative analgesic consumption [37]. According to the mechanisms of action of the two drugs, duloxetine has a central nervous system desensitization effect and is more suitable for patients with neuropathic pain. For cases other than central sensitization, pregabalin may be more appropriate. However, postoperative pain after TKA is both neurotic and nociceptive, and a multimodal analgesic regimen combining the two drugs may be more effective than using them alone. However, this hypothesis still needs to be studied. Unfortunately, there are no clear reports comparing the adverse reactions of these two drugs.

This study’s main strength is its strict inclusion and exclusion criteria; that is, only RCTs using duloxetine before and after surgery are included, and the overall quality of the studies is high. Secondary strengths are as follows: this is the first paper to date to perform a meta-analysis on this topic; we conducted adequate subgroup analyses and sensitive analysis to clarify the robustness of the primary outcome; reliable validation tools also assessed the quality of evidence for all outcomes.

Some limitations should be clarified before interpreting our findings. First, in addition to differences in duloxetine dose, the timing of preoperative and postoperative duloxetine use was not entirely consistent across studies, which is one of the sources of heterogeneity in outcomes. Second, postoperative analgesia and intraoperative analgesia (e.g., peripheral nerve blocks, periarticular injections) also varied widely among studies, but subgroup analyses were difficult to perform. Third, the Ho et al. study did not directly show the dispersion of effects, which we attempted to address by contacting the authors but never received a response [19]. Therefore, we can only estimate the data through relevant statistical analysis. Finally, our included studies and sample size still need to be improved, and more multicenter RCTs are required to confirm our findings. Furthermore, future research could assess the length of stay and patient satisfaction. Of course, the dose-response effect of duloxetine also deserves more attention.

## Conclusion

In the present study, duloxetine reduced overall opioid consumption in the perioperative period and relieved pain levels within three weeks after surgery without increasing the risk of adverse drug events. Duloxetine can be part of a multimodal pain management regimen in patients with THA and TKA.

### Electronic supplementary material

Below is the link to the electronic supplementary material.

Supplementary Material 1

## Data Availability

The original contributions presented in the study are included in the article/ Supplementary data; further inquiries can be directed to the corresponding author.

## Abbreviations

THA: Total hip arthroplasty
TKA: Total knee arthroplasty
RR: Risk ratio
CI: Confidence interval
SMD: Standardized mean difference
RCTs: Randomized controlled trials
